# Corticospinal and Reticulospinal Contacts on Cervical Commissural and Long Descending Propriospinal Neurons in the Adult Rat Spinal Cord; Evidence for Powerful Reticulospinal Connections

**DOI:** 10.1371/journal.pone.0152094

**Published:** 2016-03-21

**Authors:** Emma J. Mitchell, Sarah McCallum, Deborah Dewar, David J. Maxwell

**Affiliations:** Spinal Cord Group, Institute of Neuroscience and Psychology, College of Medicine, Veterinary Medicine and Life Sciences, University of Glasgow, Glasgow, G12 8QQ, United Kingdom; Tokyo Medical and Dental University, JAPAN

## Abstract

Descending systems have a crucial role in the selection of motor output patterns by influencing the activity of interneuronal networks in the spinal cord. Commissural interneurons that project to the contralateral grey matter are key components of such networks as they coordinate left-right motor activity of fore and hind-limbs. The aim of this study was to determine if corticospinal (CST) and reticulospinal (RST) neurons make significant numbers of axonal contacts with cervical commissural interneurons. Two classes of commissural neurons were analysed: 1) local commissural interneurons (LCINs) in segments C4-5; 2) long descending propriospinal neurons (LDPNs) projecting from C4 to the rostral lumbar cord. Commissural interneurons were labelled with Fluorogold and CST and RST axons were labelled by injecting the b subunit of cholera toxin in the forelimb area of the primary somatosensory cortex or the medial longitudinal fasciculus respectively. The results show that LCINs and LDPNs receive few contacts from CST terminals but large numbers of contacts are formed by RST terminals. Use of vesicular glutamate and vesicular GABA transporters revealed that both types of cell received about 80% excitatory and 20% inhibitory RST contacts. Therefore the CST appears to have a minimal influence on LCINs and LDPNs but the RST has a powerful influence. This suggests that left-right activity in the rat spinal cord is not influenced directly via CST systems but is strongly controlled by the RST pathway. Many RST neurons have monosynaptic input from corticobulbar pathways therefore this pathway may provide an indirect route from the cortex to commissural systems. The cortico-reticulospinal-commissural system may also contribute to functional recovery following damage to the CST as it has the capacity to deliver information from the cortex to the spinal cord in the absence of direct CST input.

## Introduction

Descending systems have a crucial role in the selection of motor output patterns by influencing the activity of interneuronal networks in the spinal cord. Although some descending systems have direct actions on motoneurons [1] most of their actions are mediated via interneurons [1, 2]. Key components of motor networks are commissural interneurons (CINs) which project to the contralateral grey matter and coordinate left-right fore and hind-limb motor activity. Two pathways known to have a powerful influence on motor networks are the corticospinal tract (CST) and the reticulospinal tract (RST) [1]. In the rat the CST projects directly from the sensorimotor cortex and terminates principally in the deep dorsal horn and intermediate grey matter [3–5] whereas the RST originates from medullary and pontine nuclei [6–9] and terminates principally within the intermediate grey matter and lamina (VIII) [5, 10, 11]. Components of this pathway are monosynaptically activated by corticobulbar pathways and form an indirect cortico-reticular pathway to the spinal cord [12, 13]. In this study we investigated the connectivity of CST and RST axons with two classes of CINs located within the cervical spinal cord; those with local intrasegmental (LCINs) projections and long-range descending propriospinal neurons (LDPNs) that project to the contralateral or ipsilateral lumbar cord. Both LCINs and LDPNs are known to make monosynaptic connections with contralateral motoneurons [13, 14] and therefore have the capacity to influence directly motor activity in the contralateral side of the cord.

Approximately ten per cent of CINs of the lumbar spinal cord can be classed as LCINs with axons confined to a single segment [15]. Lumbar LCINs in the rat are concentrated in laminae VII, VIII and X [16] but, despite the known importance of LCINs in coordinating activity of left-right locomotor activity [17], equivalent cells have yet to be investigated in the rat cervical cord. Much of our knowledge of CINs is derived from studies of the cat lumbar spinal cord [15, 18–20] but there is considerably less information available on CINs located within the cervical spinal cord. An anatomical tracing study in cat cervical cord revealed that most LCINs are located in medial lamina VIII [21]. One group of CINs is located in the upper cervical cord (C1 to C3). These cells project to contralateral neck motor neurons located a few segments rostral or caudal from their somata and participate in bilateral vestibulocollic reflexes [21, 22]. Another group of cervical CINs in the cat are found segments C3 and C4 and project to contralateral forelimb motor neurons (C6 to Th1); these cells are active during targeted-reaching and may provide postural stability to the contralateral limb when the ipsilateral limb is lifted [23]. In monkeys there is evidence that a group of CINs exists in caudal segments of the cervical cord that may have an important role in reaching and control of coordinated hand movements [24].

A further population of cervical CINs in the rat are long-range descending propriospinal neurons (LDPNs) that project to the rostral lumbar cord and are present in all cervical segments [14, 25–28]. Approximately half of these cells are commissural and project to contralateral lumbar segments. Although the specific role of contralaterally versus ipsilaterally projecting LDPNs is unknown, it is well established that propriospinal circuitry between cervical and lumbar enlargements is crucial for the coupling of fore- and hindlimb activity [28, 29] and it is likely that these different subpopulations of LDPNs contribute to the adjustment of hindlimb position and posture during specific movements of the forebody [30]. In the cat, contralaterally and ipsilaterally projecting LDPNs can be monosynaptically excited by stimulation of the contralateral pyramid and by RST axons within the medial longitudinal fasciulus (MLF) [31, 32]. A recent trans-synaptic viral tracing study in mice [26] revealed that both contralaterally (commissural) and ipsilaterally projecting LDPNs make monosynaptic connections with lumbar motor neurons. Although some of these cells received contacts from CST axons, many cells received no contacts and any contacts on LDPNs were sparse. Given this contradictory evidence it is important to determine to what extent CST axons make contact with LDPNs.

An additional reason for investigating these putative connections is that they may have the capacity to act as ‘detour’ circuits following unilateral damage to the CST following stroke or injury. Detour circuits are pre-existing pathways that assume a new or greater role to compensate for the loss of direct corticospinal input to the spinal cord [33]. Commissural interneurons are strong candidates for components of such pathways as they have the capacity to convey information from supraspinal structures to the denervated side of the spinal cord.

In rats, the principal crossed component of the CST descends within the ventral region of the dorsal white columns[3, 34, 35] and gives rise to numerous collateral branches that terminate in the dorsal horn and intermediate grey matter [3, 4]. For this reason we injected tracer into the forelimb region of the sensorimotor cortex and focused upon LCINs and LDPNs that were located in regions of the grey matter where there was significant overlap with CST terminations. In addition, although there are several components of the RST, axons projecting via the MLF were labelled because electrophysiological studies demonstrate that activation of RST fibres within this region produces profound effects on networks involved in motor control [19]. RST axons have extensive bilateral projections to the spinal cord [5, 11] and therefore contacts on LDPNs located on both sides of the spinal cord were characterised to examine whether the RST differentially targets contralaterally (commissural) versus ipsilaterally projecting LDPNs. We focused on cells within C4 and C5 segments because motor nuclei supplying proximal and distal muscles of the forelimb are present within these segments [36]. The overall aim of the study was to determine if CST and/or RST terminals form substantial numbers of contacts with CINs and thus to test the hypothesis that cervical CINs are influenced by monosynaptic action of CST and RST pathways.

## Materials and Methods

All animal procedures were carried out under licence from the UK Home Office, in accordance with the Animals (Scientific Procedures) Act (1986) and were approved by the Glasgow University Animal Welfare and Ethical Review Panel. Twelve adult male Sprague Dawley rats (Harlan, Bicester, UK) weighing between 250-350g were used in this study. Six rats received Fluorogold (FG) injections into the right intermediate grey matter of the cervical cord (C4/C5; to retrogradely label LCINs) and the b subunit of cholera toxin (CTb) was injected either into the right forelimb motor cortex or the MLF (3 rats in each group). A further six rats received FG injections into the right intermediate grey matter of the lumbar cord (L1/L2; to retrogradely label LDPNs) and CTb was injected either into the right forelimb motor cortex or the MLF (3 rats in each group). All animals were induced and maintained with isofluorane (up to 4% in oxygen) anaesthesia during surgical procedures.

### Anterograde Labelling of Axonal Terminals

Following induction of anaesthesia rats were transferred to a stereotaxic frame and a midline incision was made to expose the surface of the skull. In rats where the forelimb motor cortex was targeted, Bregma was used as a stereotaxic reference point; whereas in rats where the MLF was targeted the Interaural Line was used. The stereotaxic coordinates for each target region are listed in Table 1. The coordinates for the forelimb area were based on those defined by Neafsey et al [37] and the coordinates for the MLF were obtained from the stereotaxic atlas of Paxinos and Watson [38]. A drill (World Precision Instruments, Inc.) was used to expose the surface of the brain. For forelimb area injections, burr holes were drilled at two locations, then subsequently connected. For MLF injections, one burr hole was drilled. A micropipette containing 1% CTb (Sigma-Aldrich Co., Poole, UK) in distilled water was inserted into the brain and CTb solution was expelled by using an air pressure device (1–5 pound-force per square inch (psi); Pico-injector, World Precision Instruments, USA). Four 200nl injections were administered for forelimb area labelling and a single 200nl injection was administered for MLF labelling. For each injection the needle remained in place for 5 min to prevent backflow. The scalp was then stitched (4–0 silk sutures) and 2ml 0.9% saline was injected subcutaneously. The isofluorane was switched off and the animal was allowed to breathe 100% O_2_. Once the rat regained the foot-pinch withdrawal reflex, it was transferred to a recovery cage.

**Table 1:** Stereotaxic coordinates used to label corticospinal (CST) and reticulospinal (RST) axons. Based on Paxinos & Watson, [38].

**Descending system**	**Target**	**Bregma coordinates, mm**		
		**AP**	**ML**	**DV**
**CST**	**Forelimb sensorimotor cortex**	+1	-3	-1.5
		-0.5	-1.5	-1.5
**RST**		**Interaural coordinates, mm**		
		**AP**	**ML**	**DV**
	**Medial longitudinal fascicle**	-3.8	+0.1	+1.0

### Retrograde Labelling of Propriospinal and Commissural Interneurons in the Cervical Spinal Cord

Following a two day recovery period from surgery, animals were anaesthetized again as described above. To label short-range cervical LCINs animals were placed in a stereotaxic frame and the C4/5 segment was targeted by counting down from the prominent C2 spinous process. To label LDPNs in the cervical spinal cord animals were placed in a spinal frame and the L1 segment was targeted by counting down from the point of attachment of the lowest rib at T13. A dorsal midline incision was made extending from Th10 to L3 or C1 to C6. A small burr hole (1mm diameter) was then made on the right side adjacent to the midline in the laminar surface to expose the dorsal surface of the L1/2 or C4/5 segment of the spinal cord. A small hole was made in the pia mater with a fine syringe needle to prevent dimpling of the spinal cord during micropipette insertion. A micropipette (20μm diameter) containing FluoroGold (FG, 4%, in distilled water; Flourochrome, LLC, USA) was inserted into the spinal cord to a depth of ~1.5mm from the surface at an angle of 15° and 50nl was pressure-injected. The micropipette was held in place for 5min. The wound was sutured and each animal received a subcutaneous injection of saline (2ml, 0.9%) and Buprenorphine (0.1mg/100g) and Carprofen (5mg/100g; Reckitt Benkiser Healthcare, Dumfries, UK). Following withdrawal of anaesthesia animals regained consciousness and their health status was monitored throughout the survival period by trained staff of the Central Research Facility of the University of Glasgow. Animals were housed in pairs in standard rat cages and provided with food and water *ad libitum*. All animals recovered uneventfully.

### Perfusion Fixation

Seven days after the initial injections, animals were anaesthetized with Pentobarbitone (1ml/200g, intraperitoneally; Sigma-Aldrich, UK) and perfused transcardially with fixative (4% formaldehyde in 0.1M phosphate buffer, pH 7.4). The vertebral column was dissected to expose the cervical or lumbar spinal cord and segments were identified by counting dorsal roots *in situ*. Individual segments were then removed, post-fixed for 8 hours at 4°C and were cut into 60μm thick transverse sections with a Vibratome (Oxford Instruments, Technical Products International, Inc., USA). All sections were treated with an aqueous solution of 50% ethanol for 30 min to aid complete antibody penetration. Brains were fixed in the same solution except sucrose (3g/10ml) was added to the fixative. Brains were sectioned coronally (90 μm thick) with a freezing microtome.

### Tissue Processing

Cervical sections containing commissural and descending propriospinal neurons were reacted with antibodies to identify CTb and FG. Sections containing CST axons were also reacted for the vesicular glutamate transporter 1 (VGLUT1) and those containing RST axons were reacted either for vesicular glutamate transporter 2 (VGLUT2) or the vesicular GABA transporter (VGAT). All incubations with primary antibodies were carried out for 48 hours (**See** Table 2 for details). Subsequently sections were incubated in secondary antibodies coupled to fluorophores for 3 hours and mounted on glass slides with anti-fade medium, (Vectashield; Vector Laboratories, Peterborough, UK). Cervical and lumbar spinal injection sites containing FG were examined with UV epifluorescence and photographed whereas cortical and MLF injection sites were visualized by using 3, 3’-diaminobenzidine (DAB). Sections were incubated in goat anti-CTb for 48 hours followed by biotinylated anti-goat IgG for 3 hours at room temperature. They were then incubated in avidin-horseradish peroxidase (HRP) for 1 hour and hydrogen peroxide plus DAB was applied for a period of approximately 15 min to reveal immunoreactivity.

**Table 2:** Antibodies used in the study: Mo = mouse; gp = guinea pig; rb = rabbit; gt = goat Rh.Red = rhodamine red.

Group	Primary antibody combination	Concentration	Supplier/ catalogue No	Secondary antibody combination	Dilution	Sequential reaction
**1**	gt. CTb	1:5000	List Biological Laboratories, Campell, CA #703	Biotinylated IgG	1:500	Avidin HRP (1:1000) + DAB
**2**	gt. CTb	1:5000	List Biological Laboratories, Campell, CA #703	Rh. Red	1:100	
	rb. FG	1:5000	Chemicon/Millipore, CA, USA AB153	Alexa 488	1:500	
	gp. VGLUT1	1:5000	Millipore, Harlow, UK AB5905	Dylight 649	1:500	
**3**	gt. CTb	1:5000	List Biological Laboratories, Campell, CA #703	Rh. Red	1:100	
	rb. FG	1:5000	Chemicon/Millipore, CA, USA AB153	Alexa 488	1:500	
	gp. VGLUT2	1:5000	Chemicon/Millipore, CA, USA AB2251	Dylight 649	1:500	
**4**	gt. CTb	1:5000	List Biological Laboratories, Campell, CA #703	Rh. Red	1:100	
	rb. FG	1:5000	Chemicon/Millipore, CA, USA AB153	Alexa 488	1:500	
	mo. VGAT	1:1000	Synaptic Systems GmbH,Göttingen, Germany 131 001	Dylight 649	1:500	

### Confocal Microscopy, Reconstructions and Analysis

Immunoreactive sections containing labelled cells and terminals were scanned with a three-colour channel confocal laser scanning microscope (Radiance 2100, Biorad, UK). Systematic low power scans (x20 objective, zoom factor of 1) were initially performed to assess the laminar location of neurons and to also locate neurons with labelled terminals in their vicinity. Neurons for analysis were selected according to the following criteria: 1) They had dendritic arbors with at least second order dendrites; 2) They were located within CST and RST termination zones. Cells were analysed from 3–5 sections per experimental condition per rat. Selected neurons were scanned at 40x oil immersion with a zoom factor of 2 at 0.5μm increments and reconstructed three dimensionally from series of single optical sections (approximately 100 per cell) using Neurolucida 9.14.3 software (MBF Bioscience, MicroBrightField, Inc.). Cell bodies and dendritic processes were drawn initially and then contacts were plotted onto the drawings. Contacts were defined as being in close apposition to neuronal processes in the same focal plane with no intervening black pixels. For a given cell the total dendritic surface area (μm^2^) was recorded directly from Neurolucida 9.14.3 software. The surface area of the soma was calculated by firstly measuring the perimeter from the projected confocal image using Image J (1.43, National Institutes of Health, USA) then calculating the surface area of an equivalent sphere (radius (μm) = perimeter/(2π) and surface area (μm^2^) = 4πr^2^). Datum was the total number of contacts per unit area of neuronal surface (100μm^2^). Data are expressed as mean ± standard deviation (SD).

## Results

### Injection Sites

#### Intrasegmental commissural interneurons

**Fig 1:**
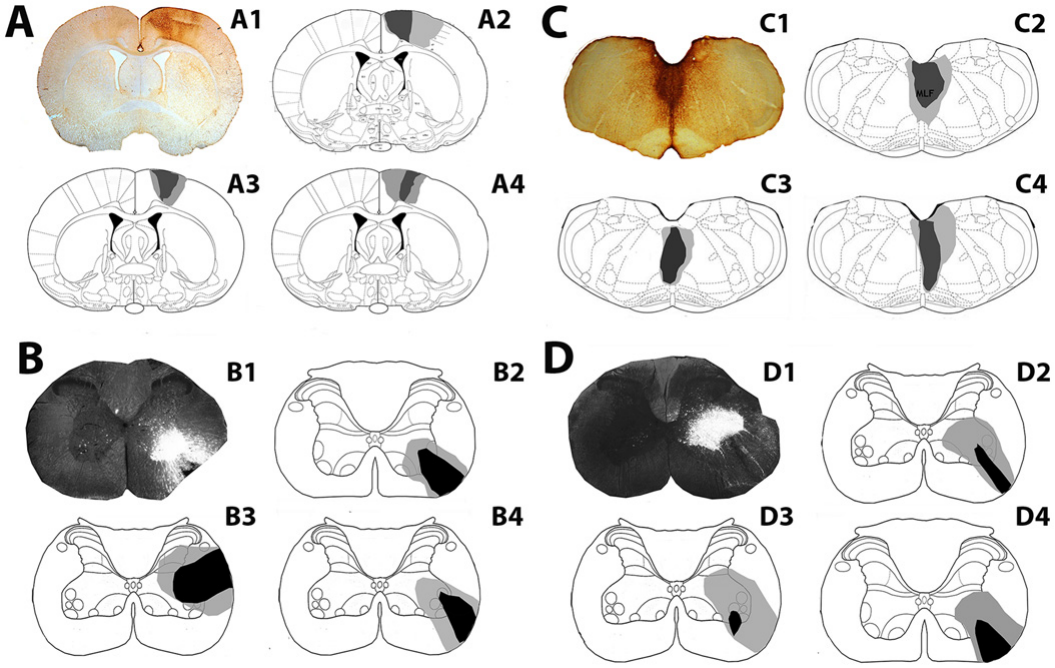
Injection sites for experiments with cervical local commissural interneurons for individual rats used in the study. A: A1 Representative CTb injection site showing immunoperoxidase reaction in the cortex. A2-A4 are reconstructions of injection sites for 3 rats. Injections are focused on M1 with spread into M2 and S1. B: B1 is a representative fluorescence/dark field micrograph showing a FG injection site within a transverse section of the cervical cord (C4); B2-B4 are reconstructions of spinal injection sites for cortical experiments for the rats shown in A. C: C1 is a CTb injection site in the medial longitudinal fasciculus (MLF); C2-C4 are reconstructions of MLF injection sites for 3 rats. D: D1 is a representative fluorescence/dark field micrograph showing a FG injection site within a transverse section of the C5 Segment; D2-D4 are reconstructions of spinal injection sites for MLF experiments for rats shown in C. The darkest shading represents the core of the injection and lighter shading represents spread beyond the core. Brain and spinal templates are taken from the atlas of Paxinos & Watson, [38].

CTb injection sites within the cortex and corresponding FG injection sites in the cervical cord (C4/C5) for each rat are shown in Fig 1A and 1B. The CTb injection sites were focussed within the forelimb regions of the primary and secondary motor cortex and the adjacent primary sensory cortex and were confined to the cortex of the right hemisphere. FG injections were confined to C4/C5; for two rats, the core of the FG injection site was within the ventrolateral funiculus and for one rat the core was in the intermediate grey matter. For all three rats, there was considerable spread of FG within the grey matter ipsilateral to the injection site but there was no spread of FG into the contralateral grey matter. Fig 1C and 1D show CTb injection sites within the MLF and the corresponding FG injection sites in the cervical cord (C4/C5). In the MLF, CTb labelling was visualised bilaterally and there was some spread of CTb into the raphe obscurus and tectospinal tract. FG injections were confined to C4/C5; for all three rats, the core of the FG injection site was within the ventrolateral funiculus and there was considerable spread of FG within the grey matter ipsilateral to the injection site but no spread of FG into the contralateral grey matter.

#### Long descending propriospinal neurons

**Fig 2:**
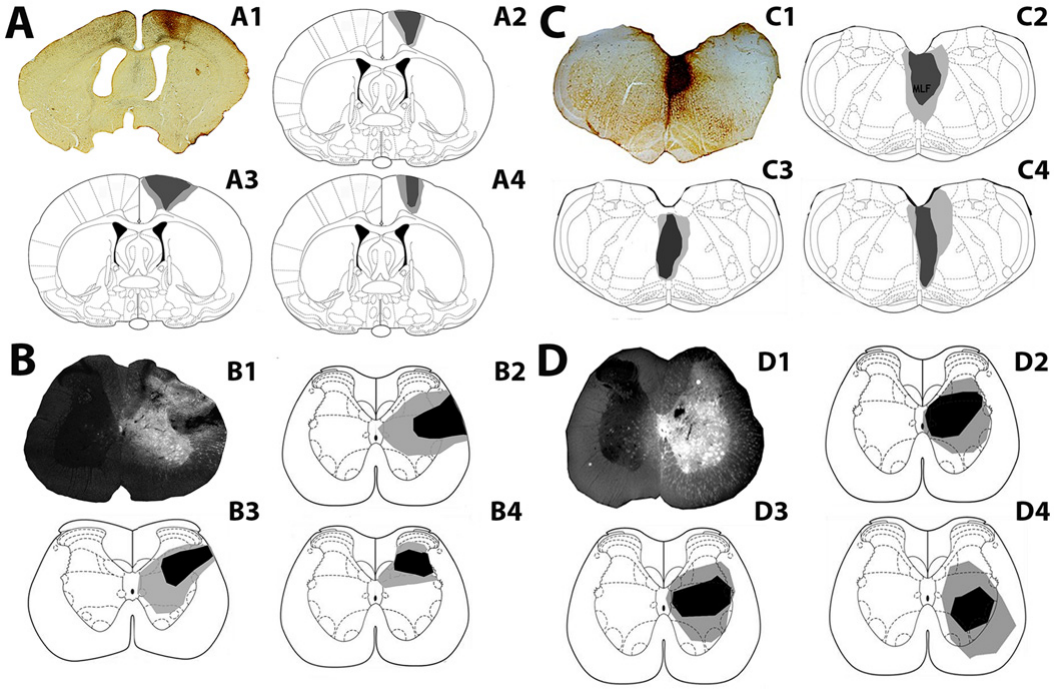
Injection sites for experiments with long descending propriospinal interneurons for individual rats used in the study. A: A1 is a representative CTb injection site showing immunoperoxidase reaction in the cortex; A2-A4 are reconstructions of cortical injection sites for 3 rats. Injections are focused on M1 with spread into M2 and S1. B: B1 is a representative fluorescence/dark field micrograph showing a FG injection site within a transverse section of the lumbar spinal cord (L2); B2-B4 are reconstructions of spinal injection sites for cortical experiments for rats shown in A.C: C1 is a CTb injection site in the medial longitudinal fasciculus (MLF); C2-C4 are reconstructions of MLF injection sites for 3 rats. D: D1 is a representative fluorescence/dark field micrograph showing a FG injection site within a transverse section of the L2 segment; D2-D4 are reconstructions of spinal injection sites for MLF experiments for the rats shown in C. The darkest shading represents the core of the injection and lighter shading represents spread beyond the core. Brain and spinal templates are taken from the atlas of Paxinos & Watson [38].

Injection sites in the cortex and corresponding FG injection sites in the lumbar cord (L1/L2) for each rat are shown in Fig 2A and 2B. Injection sites were focussed within the primary and secondary motor cortex and the adjacent primary sensory cortex as described above for LCIN experiments. FG injections were within L1/L2 segments. Spread of FG was observed within the grey matter ipsilateral to each injection but there was no spread to the contralateral grey matter. Fig 2C and 2D show injection sites within the MLF and corresponding FG injection sites in the lumbar cord. The micropipette was placed in the right side of the MLF but CTb labelling was visualised bilaterally. The FG injections into the lumbar spinal cord were found within L1/L2, with the injection site centred in the intermediate grey matter.

### Laminar Distribution Pattern of LCINs and LDPNs in Relation to CST and RST Terminals in the Cervical Cord

**Fig 3:**
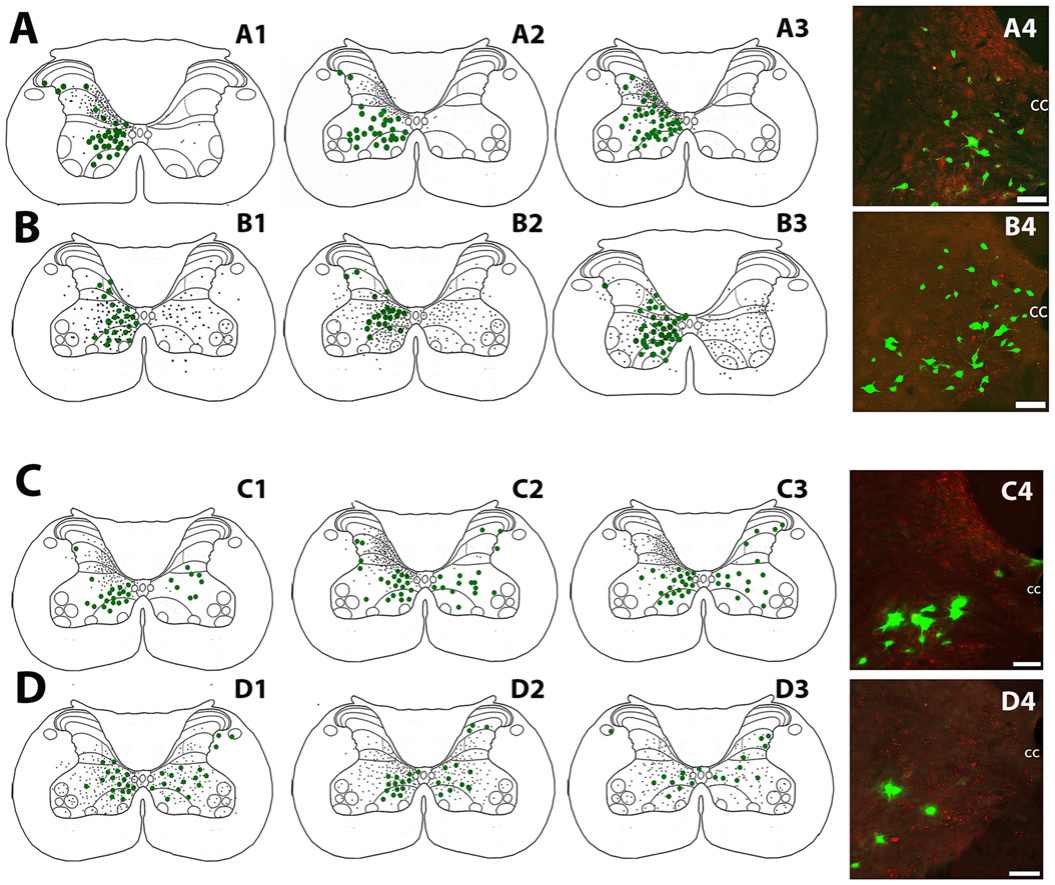
Distribution of local commissural interneurons and long descending propriospinal interneurons in relation to corticospinal and reticulospinal terminations in the cervical spinal cord for each rat used in the study. Cells are represented as green dots and axon terminals as small black dots on spinal outline diagrams of the C4/5 cervical segments. A1-4: Distribution of local commissural interneurons (LCINs) and corticospinal terminals (CST). A4 is a projected confocal image showing the distribution of LCINs (green) CST terminals (red) adjacent to the central canal. B1-4: Distribution of LCINs and reticulospinal (RST) terminals. B4 is a projected confocal image showing LCINs and RST terminals. C1-4: Distribution of long descending propriospinal neurons (LDPNs) and CST terminals. C4 is a projected confocal image showing LDPNs and CST terminals. D1-4: Distribution of LDPNs and RST terminals. D4 is a projected confocal image showing LDPNs and RST terminals. CC = central canal in confocal images. Scale bars = 50μm.

Fig 3A and 3B show the laminar distribution pattern of LCINs in relation to CST and RST terminals in segments C4 or C5 for all 6 rats. The distribution of FG-labelled LCINs within the grey matter was similar for all rats. They were distributed throughout laminae VI to VIII and within lamina X. CTb labelled CST terminals were located in the grey matter contralateral to the cortical injection site and contralateral to the cervical spinal injection. Although the majority of CST terminals were located dorsally to LCINs (in laminae IV to VI), a moderate number of terminals were present in laminae VI to VII. CTb-labelled RST terminals were distributed bilaterally and were numerous through the deep dorsal horn, intermediate grey matter and ventral horn.

Fig 3C and 3D shows the laminar distribution pattern of LDPNs in relation to CST and RST terminals in segment C5 for all 6 rats. The distribution of FG-labelled LDPNs within the grey matter was similar for all animals. The largest numbers of cells were found in laminae VII and VIII, both contralateral and ipsilateral to the lumbar injection site; however the contralateral cells tended to be concentrated within the medial region of laminae VII whereas ipsilateral cells were more evenly distributed throughout the intermediate grey matter. CST and RST terminals were distributed in an identical pattern to that described above for experiments with cervical commissural interneurons.

### CST and RST Contacts on LCINs

**Fig 4:**
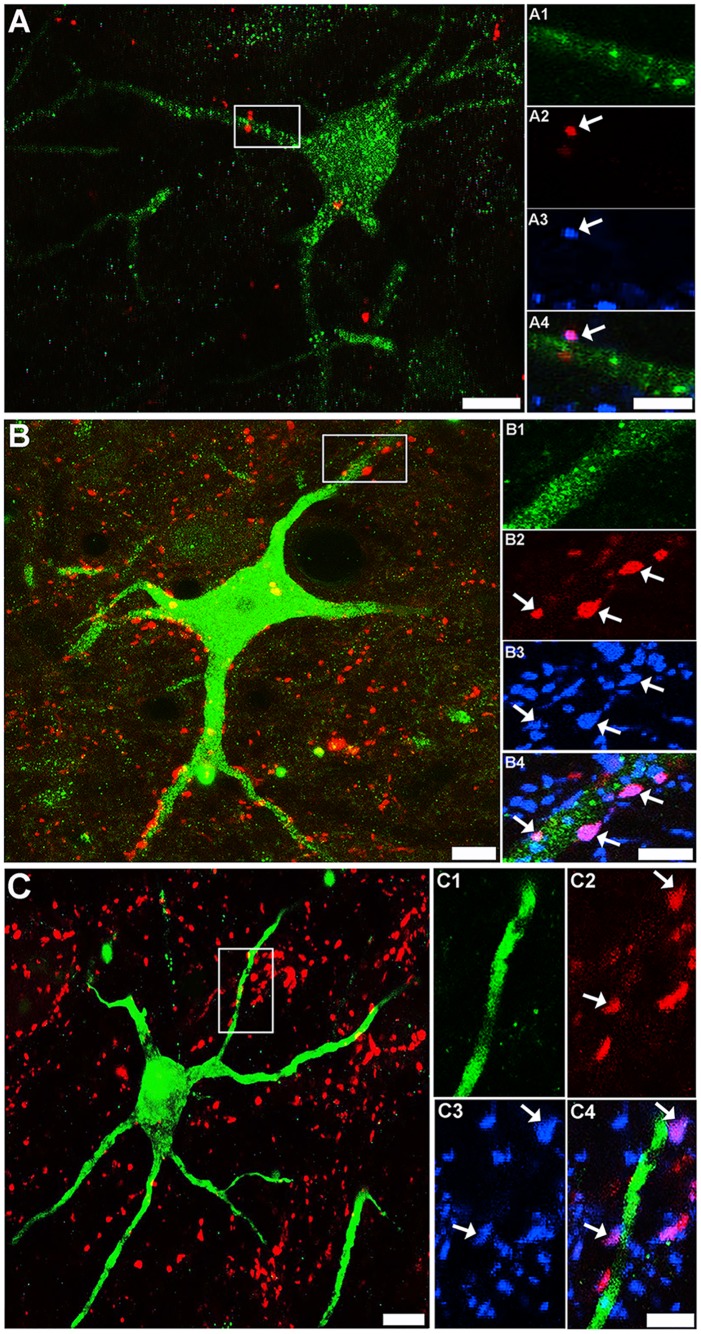
Confocal images of corticospinal and reticulospinal contacts on local commissural interneurons. A: a projected confocal microscope image of a FG-labelled LCIN (green) and CTb-labelled CST terminals (red) taken from the medial region of lamina VII in segment C4. Scale bar = 20μm. Insets A1—A4 are single optical sections that correspond to the region demarcated in A showing a single CST contact on this cell (arrow). A1 immunoreactivity for FG; A2 immunoreactivity for CTb; A3 immunoreactivity for VGLUT1. A4 is a merged image of A1-3. Scale bar = 5 μm. B: a projected confocal microscope image of a FG-labelled cell (green) and CTb-labelled RST terminals (red) taken from the medial region of lamina VII in segment C5. Scale bar = 20μm. Insets B1—B4 are single optical sections that correspond to the region demarcated in B. B1 immunoreactivity for FG; B2 immunoreactivity for CTb; B3 immunoreactivity for VGLUT2. A4 is a merged image of B1-3: the arrows indicate VGLUT2 positive contacts. Scale bar = 5 μm. C: a projected confocal microscope image of a LCIN (green) and CTb-labelled RST terminals (red) in segment C5. Scale bar = 20μm. C1—C4 are single optical sections that correspond to the region demarcated in C. C1 immunoreactivity for FG; C2 immunoreactivity for CTb; C3 immunoreactivity for VGAT. A4 is a merged image of C1-3: the arrows indicate VGAT-positive contacts. Scale bar = 5 μm.

A total of 39 retrogradely labelled cells were scanned with the confocal microscope and reconstructed to determine the extent of CST contacts on LCINs. On inspection of the first few cells, it was found that although CTb-labelled terminals were present within the vicinity of the cells, they rarely established contacts with them. Four cells with detected contacts were re-scanned in order to confirm that the terminals contacting them contained VGLUT1. Fig 4A shows an example of a LCIN with a CTb-labelled contact that was immunoreactive for VGLUT1. Thirty one cells were reconstructed to examine excitatory RST contacts on LCINs. A considerable number of CTb-labelled RST terminals were present in the vicinity of CINs and many of these terminals were found to establish excitatory (VGLUT2 immunoreactive) contacts with them. Fig 4B shows an example of a cell with numerous excitatory (VGLUT2 immunoreactive) RST contacts. As summarised in Table 3, the majority of RST contacts to LCINs (78.8 ± 4.0%) were immunoreactive for VGLUT2. A further 21 cells were examined for inhibitory RST contacts (Fig 4C). As shown in Table 3 a sizeable minority of RST contacts on LCINs (19.8 ± 2.4%) were immunoreactive for VGAT.

**Table 3:** Excitatory and inhibitory contacts on local commissural interneurons. All cells were located within segments C4-5 and a minimum of three sections was analysed per experimental condition per rat.

**RAT**	**No. cells reconstructed**	**CTb contacts (total)**	**VGLUT2**^**+CTb**^	**% VGLUT2**
1	11	136	108	79.4
2	10	97	80	82.5
3	10	196	146	74.5
**Mean ± SD**				**78.8±4.0**
			**VGAT** ^**+CTb**^	**% VGAT**
1	8	72	13	18.1
2	5	96	18	18.8
3	7	133	30	22.6
**Mean ± SD**				**19.8±2.4**

### CST and RST Contacts onto LDPNs

**Fig 5:**
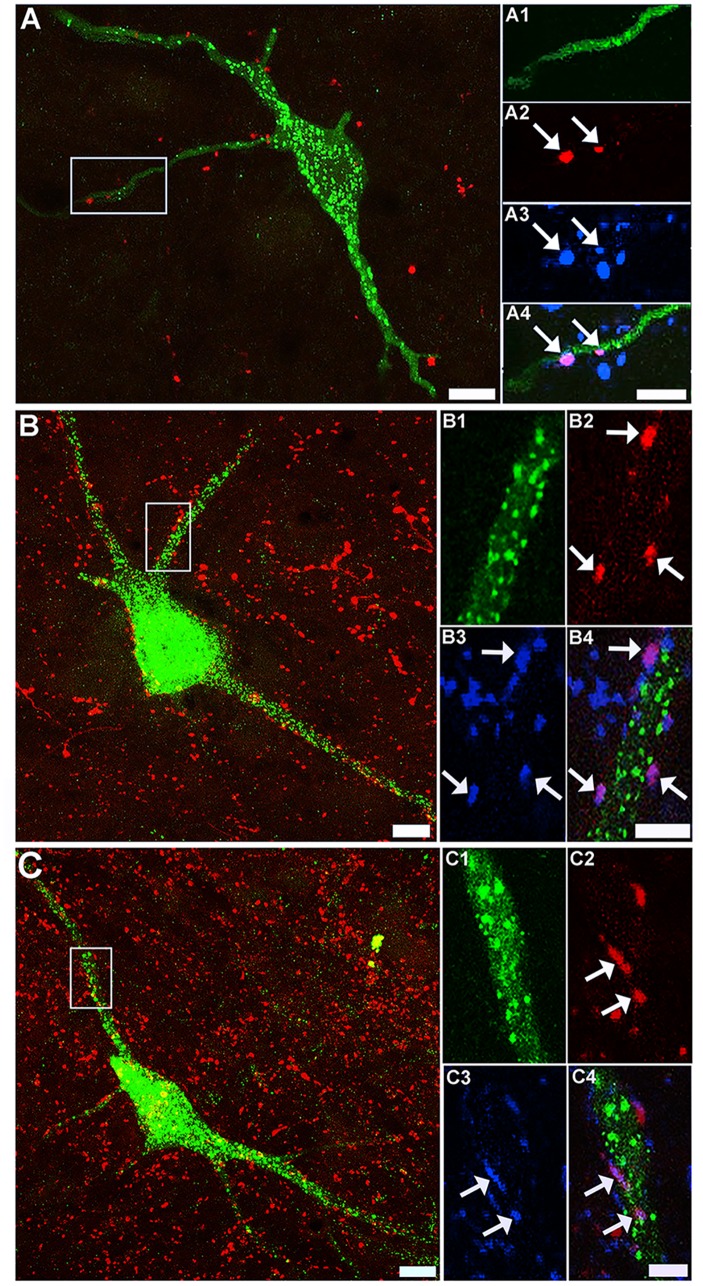
Confocal images of corticospinal and reticulospinal contacts on long descending propriospinal neurons. A: a projected confocal microscope image of a FG-labelled LDPN (green) and CTb-labelled CST terminals (red) taken from the medial region of lamina VII (contralateral to the L1/L2 injection site; segment C5). Scale bar = 20μm. Insets A1—A4 are single optical sections that correspond to the region demarcated in A showing two CST contact on this cell (arrows). A1 immunoreactivity for FG; A2 immunoreactivity for CTb; A3 immunoreactivity for VGLUT1. A4 is a merged image of A1-3. Scale bar = 5 μm. B: a projected confocal microscope image of a FG-labelled cell (green) and CTb-labelled RST terminals (red) taken from the medial region of lamina VII (contralateral to lumbar injection; segment C5). Scale bar = 20μm. Insets B1—B4 are single optical sections that correspond to the region demarcated in B. B1 immunoreactivity for FG; B2 immunoreactivity for CTb; B3 immunoreactivity for VGLUT2. B4 is a merged image of B1-3: the arrows indicate VGLUT2-positive contacts. Scale bar = 5 μm. C: a projected confocal microscope image of a LDPN (green) and CTb-labelled RST terminals (red) in segment C5. Scale bar = 20μm. C1—C4 are single optical sections that correspond to the region demarcated in C. C1 immunoreactivity for FG; C2 immunoreactivity for CTb; C3 immunoreactivity for VGAT. C4 is a merged image of C1-3: the arrows indicate VGAT-positive contacts. Scale bar = 5 μm.

In total, 32 retrogradely labelled cells were scanned and reconstructed to examine CST contacts on LDPNs. A moderate number of CTb-labelled CST terminals were present within the vicinity of contralaterally-projecting LDPNs but few of these terminals established contacts with labelled cells. Fig 5A shows an example of a commissural LDPN with numerous CTb-labelled terminals within its immediate territory, but only 2 of the terminals were found to establish contacts with the cell. All CTb-labelled contacts to commissural LDPNs were immunoreactive for VGLUT1. A total of 36 cells (18 contralateral and 18 ipsilateral to the L1/L2 injection sites) were reconstructed and examined for excitatory RST terminals. A large number RST terminals were found close to contralaterally and ipsilaterally projecting LDPNs and many of these terminals established excitatory (VGLUT2 immunoreactive) contacts with the cells (Fig 5B). The majority of RST contacts to both contralateral (78.9 ±4.7%) and ipsilateral (73.4 ± 8.0%) LDPNs were immunoreactive for VGLUT2 (Table 4). In all, 33 cells (20 contralateral and 13 ipsilateral to the L1/L2 injection site) were examined for inhibitory RST contacts (Fig 5C). A sizeable minority of RST contacts on contralateral (20.7 ± 2.7%) and ipsilateral (19.7 ± 2.3%) LDPNs were immunoreactive for VGAT.

**Table 4:** Excitatory and inhibitory reticulospinal contacts on long descending propriospinal interneurons contralateral and ipsilateral to L2 injection site. All cells were located within segments C4-5 and a minimum of three sections was analysed per experimental condition per rat.

	**RAT**	**No. cells reconstructed**	**CTb contacts (total)**	**VGLUT2**^**+CTb**^	**% VGLUT2**
**Contralateral Cells**	4	7	356	283	79.5
	5	6	81	60	74.1
	6	5	84	70	83.3
**Mean ± SD**					**78.9 ±4.7**
**Ipsilateral Cells**	4	5	262	171	65.3
	5	7	102	75	73.5
	6	6	299	243	81.3
**Mean ± SD**					**73.7±8.0**
	RAT	**No. cells reconstructed**	**CTb contacts (total)**	**VGAT** ^**+CTb**^	**% VGAT**
**Contralateral Cells**	4	8	733	169	23.1
	5	5	72	14	19.4
	6	7	427	76	17.8
**Mean ± SD**					**20.1±2.7**
**Ipsilateral Cells**	4	4	32	8	22.2
	5	5	62	12	19.4
	6	4	91	16	17.6
**Mean ± SD**					**19.7±2.3**

### Quantitative Analysis of Contact Densities on LCINs

#### CST contacts

**Fig 6:**
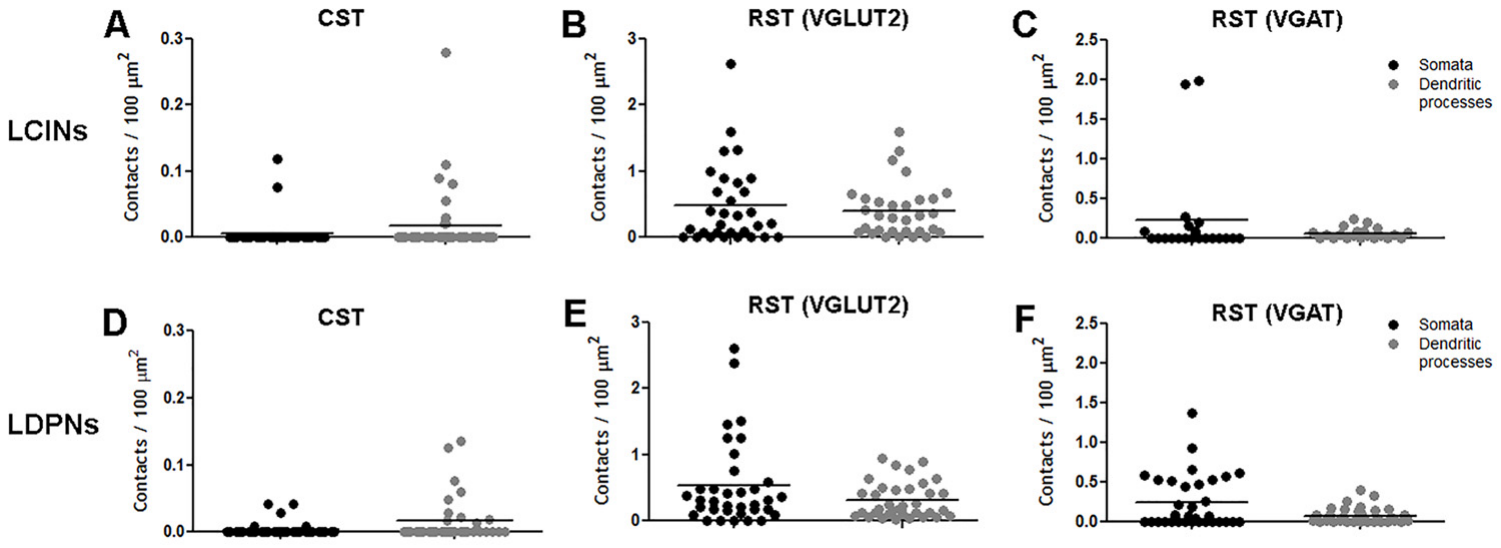
Contact densities on local commissural interneurons (LCINs) and long descending propriospinal interneurons (LDPNs) in the cervical spinal cord. Each data point represents the contact density of an individual cell. Black dots represent somatic contacts and grey dots represent dendritic contacts. Units are in contacts/100 μm^2^. CST = corticospinal tract; RST (VGLUT2) = contacts made by reticulospinal tract terminals immunoreactive for VGLUT2; RST (VGAT) = contacts made by reticulospinal tract terminals immunoreactive for VGAT. Note the difference in scale of Y axis of A and D showing paucity of CST contacts on both LCINs and LDPNs.

The mean number of CTb-labelled CST contacts per 100μm^2^ of neuronal surface for the 39 cells analysed was higher for dendritic processes (0.02 ± 0.05) when compared with somata (0.005 ± 0.02: Fig 6A).

#### Excitatory RST contacts

Contact densities for all of the 31 cells examined are shown in Fig 6B. For somata, the mean number of CTb-labelled terminals per 100μm^2^ of neuronal surface was 0.62 ± 0.63 and the mean number of CTb-labelled terminals that contained VGLUT2 (VGLUT2^+CTb^) was 0.47 ± 0.60. For the dendritic processes, the equivalent number of CTb-labelled terminals per 100μm^2^ of neuronal surface was 0.46 ± 0.43 and the mean number of CTb-labelled terminals that contained VGLUT2 was 0.40 ± 0.4.

#### Inhibitory RST contacts

The contact densities for the 21 reconstructed LCINs are shown in Fig 6C. For somata, the mean number of CTb-labelled terminals per 100μm^2^ of neuronal surface was 0.76 ± 1.32 and the mean number of CTb-labelled terminals that contained VGAT was 0.23 ± 0.59; for dendritic processes, the mean number of CTb-labelled terminals per 100μm^2^ of neuronal surface was 0.29 ± 0.35 and the mean number of CTb-labelled terminals that contained VGAT was 0.06 ± 0.07.

### Quantitative Analysis of Contacts on LDPNs

#### CST contacts

The mean number of CTb-labelled CST contacts per 100μm^2^ of neuronal surface for the 21 cells analysed was higher for the dendritic processes (0.02 ± 0.03) compared to the somata (0.005 ± 0.01) (Fig 6D).

#### Excitatory RST contacts

Contact densities for all 36 LDPNs are shown in Fig 6E. For somata, the mean number of CTb-labelled terminals per 100μm^2^ of neuronal surface was 0.75 ± 0.83 and the mean number of CTb-labelled terminals that contained VGLUT2 was 0.53 ± 0.64. For dendritic processes, the mean number of CTb-labelled terminals per 100μm^2^ of neuronal surface was 0.42 ± 0.34 and the mean number of CTb-labelled terminals that contained VGLUT2 was 0.30 ± 0.27.

#### Inhibitory RST contacts

Contact densities for the 33 reconstructed LDPNs are shown in Fig 6F. For somata, the mean number of CTb-labelled terminals per 100μm^2^ of neuronal surface was 0.99 ± 1.1 and the mean number of CTb-labelled terminals that contained VGAT was 0.24 ± 0.33. For dendritic processes, the mean number of CTb-labelled terminals per 100μm^2^ of neuronal surface was 0.48 ± 0.64 and the mean number of CTb-labelled terminals that contained VGAT was 0.08 ± 0.10.

#### Commissural versus ipsilaterally projecting LDPNs

**Fig 7:**
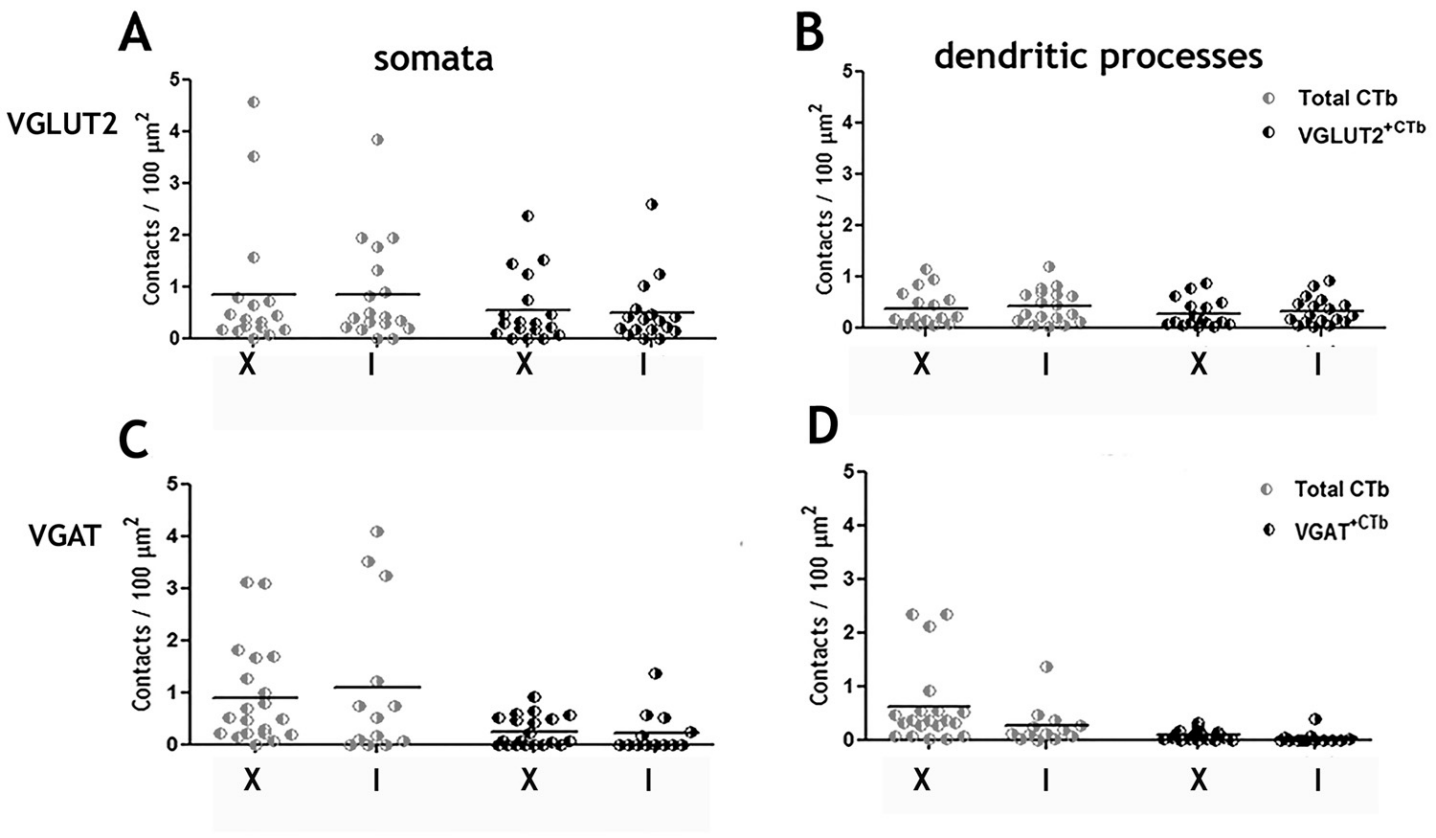
Contact densities of reticulospinal axons on ipsilateral and contralateral long descending propriospinal interneurons (LDPNs) in the cervical spinal cord. A and B show total RST contacts and VGLUT2 contacts on somata and dendrites of ipsilateral and contralateral cells to the injection site. C and D show total RST contacts and VGAT contacts on somata and dendrites of ipsilateral and contralateral cells to the injection site. Units are in contacts/100 μm^2^. VGLUT2^+CTb^ = contacts made by reticulospinal tract terminals immunoreactive for VGLUT2; VGAT^+CTb^ = contacts made by reticulospinal tract terminals immunoreactive for VGAT. I = Cells ipsilateral to L2 injection site; X = Cells contralateral to L2 injection site.

Contact densities of contralaterally (commissural) versus ipsilaterally projecting LDPNs for RST contacts on somata (Fig 7A and 7C) or dendrites (Fig 7B and 7D) were similar. This was also the case for RST/VGLUT contacts (Fig 7A and 7B) and RST/VGAT contacts (Fig 7C and 7D).

## Discussion

In this study we have shown that in the rat, CST axons originating from the forelimb region of the sensorimotor cortex form very small numbers of contacts with cervical LCINs and LDPNs whereas RST axons make approximately ten times the numbers of contacts with these cells. In the rat therefore the CST is likely to have a limited influence on commissural cells whereas the large numbers of excitatory and inhibitory RST contacts indicates that this system has powerful effects on both classes of CINs. It is well established that the cortico-reticulospinal pathway can convey indirect locomotor command signals from higher centres to the spinal cord (e.g. see Matsuyama et al.,[12]) and an increasing body of evidence also suggests that RST systems contribute to voluntary movements. For example, Soteropoulos et al., [39] reported that cells in the pontomedullary reticular formation modulate activity in response to ipsilateral fine finger movements in the macaque.

### Technical Limitations of the Study

The b subunit of cholera toxin is transported in the anterograde as well as retrograde direction [40] and it is taken up by axons of passage in the CNS in addition to axon terminals [41]. Therefore it cannot be excluded that some of the axons labelled by MLF injections were contaminated with axons from systems in addition to the RST as fibres of the medial vestibulospinal tract [42, 43] and the tectospinal tract [44] also descend within this region. However, the distribution and proportions of excitatory and inhibitory RST axons was very similar to the pattern seen in the lumbar cord following MLF injections [5] which does not have projections from these additional tracts and it is therefore probable that the majority of terminals labelled were components of the RST. Furthermore according to Shinoda et al. [45] most medial vestibulospinal tract terminations are found within motor nuclei; as sampled CINs in the present study were located principally within the intermediate grey matter it is likely that most of the contacts originated from RST axons. The MLF is a midline structure and it is not possible to make unilateral injections of tracer within it. The medial-lateral coordinate we used was +0.1 inter-aural and CTb spread contralaterally in all experiments (Figs 1 and 2); however the RST forms substantial bilateral projections within the cervical spinal cord [11] and injections within the entire MLF will label all components of the RST that pass through this structure [6]. A second complication is that in addition to RST axons, small numbers of spinoreticular cells are also labelled and collateral axons of these cells may have contaminated the sample. Angelucci et al.[46], however, reported that retrogradely transported CTb is detected in cell bodies and proximal dendrites but not collateral axons, therefore it is unlikely that this is a major issue.

A further limitation of the study is that the FG tracing method used to label interneurons does not label the most distal parts of dendritic trees and therefore the numbers of contacts on LCINs and LDPNs may have been underestimated. Nevertheless, although the estimates of contact densities may not represent absolute values the difference in the relative values of contact densities between CST and RST terminals is striking. We also contend that the majority of contacts observed are likely to be synaptic associations as the presence of vesicular transporters within terminals forming contacts confirms that they contain synaptic vesicles. Studies using combined confocal and electron microscopy[47, 48] confirm that the vast majority of contacts observed with confocal microscopy between axonal swellings and cells are indeed synaptic.

### Supraspinal Inputs to LCINs

Following unilateral FG injections into segments C4/C5, a large number of retrogradely labelled cells were found in the contralateral grey matter, concentrated within laminae VI to VIII and X. This result is consistent with data obtained from the lumbar spinal cord where the distribution of LCINs is almost identical [16, 49]. LCINs in cervical segments were found to receive almost no contacts from CST axons and it may be concluded that LCINs are not a significant target for crossed CST fibres. There is thus limited evidence for a re-crossed CST-commissural pathway in the rat and LCINs apparently have a limited function in conveying CST information to the contralateral cord. In contrast, LCINs received extensive contacts from RST axons, suggesting that the RST has a strong influence on these cells. A significant input from the RST on cervical commissural interneurons might be predicted from electrophysiological studies of LCINs in the cat lumbar spinal cord where it has been shown that cells projecting to contralateral grey matter including motor nuclei are powerfully activated monosynaptically by stimulation of the MLF [18, 20]. LCINs received both inhibitory and excitatory contacts and our data indicate that most cells have convergent excitatory and inhibitory contacts from the RST because all cells examined had significant numbers of immunonegative RST contacts regardless of whether they were stained for VGLUT2 or VGAT. RST systems therefore may facilitate or depress activity of LCINs. The functional significance of this observation remains obscure and the origin of inhibitory RST systems has not yet been established. Inhibitory RST cells are not present in pontine nuclei [9] and therefore specific medullary nuclei may give rise to these inhibitory systems. Furthermore lamina VIII CINs are a mixed population of inhibitory and excitatory cells [20] which may supress or enhance activity of contralateral motoneurons via direct and indirect actions.

### Supraspinal Inputs to LDPNs

Labelled LDPNs were found in both sides of the grey matter of the C5 segment following unilateral FG-injections into segments L1/L2. The majority of cells were concentrated within laminae VII and VIII which is consistent with previous findings [14, 25, 27]. Both contralaterally (commissural) and ipsilaterally projecting LDPNs were found to receive very few contacts from CST axons, suggesting that the CST has a limited influence on these cells. This finding is similar to that reported in a study of mice by Ni et al., [26] who observed that only half of cervical LDPNs are innervated by CST axons and if contacts were present they usually numbered only from one to two per cell. In contrast, in cats, Alstermark and colleagues [30, 50, 51] have described a group of LDPNs (mostly commissural LDPNs) with strong monosynaptic input from the CST. The contradictory findings between these studies may reflect a species difference. In cats, electrophysiological studies of the C3/C4 propriospinal system shows that the CST monosynaptically activates C3/C4 propriospinal neurons, which in turn project to forelimb motor neurons [51] but this pathway is absent in the rat where the C3/C4 propriospinal system can only be activated disynaptically by pyramidal stimulation [52]. The observation that LDPNs in the rat have sparse CST inputs and extensive RST inputs also contributes to the notion, that in rodents, the motor cortex uses RST neurons as an intermediate relay instead influencing propriospinal neurons directly. It worth noting however, that even in cats, where groups of LDPNs have strong monosynaptic input from the CST, reticular control of LDPNs is also important as stimulation of the MLF evokes monosynaptic excitatory and/or inhibitory postsynaptic potentials in the majority of LDPNs [30]. We could find no evidence for differential innervation by RST terminals of ipsilaterally or contralaterally projecting LDPNs. Both types of neuron had similar contact densities and similar ratios of inhibitory and excitatory contacts. RST cells give rise to axons that innervate several segmental levels [7, 12, 53, 54] and ramify within extensive areas of the grey matter, with some axon collaterals projecting to both sides of the cord[12]. RST systems appear to be well suited to coordinate activity on both sides of the cord (e.g. see [55]) and given that the function of LDPNs is primarily to coordinate fore and hind limb activity [28, 29] it might be surmised that the combination of these two systems provides a network that coordinates the activity of all four limbs along with the axial musculature.

### Implications for Functional Recovery following Injury to the CST

The findings of this study have implications relating to neural pathways that might underlie recovery of function following damage to the CST after stroke or injury. Following unilateral transection of the CST in primates, RST fibres descending through the MLF strengthen their output to affected flexor motor neurons [56]; thus the RST may have the capacity to replace missing actions of damaged CST fibres. Recently, it has been shown in a mouse stroke model that there is remodelling of corticobulbar systems within medullary reticular nuclei and that these changes are correlated with partial functional recovery [57]. Hence it is possible given the finding that CINs receive extensive input from RST axons that this pathway has the capacity to assume a new or greater role to compensate for the loss of direct corticospinal input to the spinal cord and therefore is a strong candidate for a “detour circuit” [33].

## References

[1] Lemon RN. Descending pathways in motor control. Annu Rev Neurosci. 2008;31:195–218. 10.1146/annurev.neuro.31.060407.125547 . 18558853

[2] Jankowska E. Interneuronal relay in spinal pathways from proprioceptors. Progress in Neurobiology. 1992;38:335–78. 1315446 10.1016/0301-0082(92)90024-9

[3] Casale EJ, Light AR, Rustioni A. Direct projection of the corticospinal tract to the superficial laminae of the spinal cord in the rat. J Comp Neurol. 1988;278(2):275–86. 10.1002/cne.902780210 . 3230165

[4] Valtschanoff JG, Weinberg RJ, Rustioni A. Amino acid immunoreactivity in corticospinal terminals. Exp Brain Res. 1993;93:95–103. 7682185 10.1007/BF00227784

[5] Du Beau A, Shakya Shrestha S, Bannatyne BA, Jalicy SM, Linnen S, Maxwell DJ. Neurotransmitter phenotypes of descending systems in the rat lumbar spinal cord. Neuroscience. 2012;227:67–79. 10.1016/j.neuroscience.2012.09.037 . 23018001

[6] Huma Z, Du Beau A, Brown C, Maxwell DJ. Origin and neurochemical properties of bulbospinal neurons projecting to the rat lumbar spinal cord via the medial longitudinal fasciculus and caudal ventrolateral medulla. Front Neural Circuits. 2014;8:40 10.3389/fncir.2014.00040 24808828 PMC4009430

[7] Reed WR, Shum-Siu A, Magnuson DS. Reticulospinal pathways in the ventrolateral funiculus with terminations in the cervical and lumbar enlargements of the adult rat spinal cord. Neuroscience. 2008;151(2):505–17. 10.1016/j.neuroscience.2007.10.025 18065156 PMC2829753

[8] Zelman FP, Leonard CM, Kow L, Pfaff DW. Ascending tracts of the lateral columns of the rat spinal cord: a study using the silver impregnation and horseradish peroxidase techniques. Exp Neurol. 1978;62:298–334. 83245 10.1016/0014-4886(78)90059-6

[9] Sivertsen MS, Perreault MC, Glover JC. Pontine reticulospinal projections in the neonatal mouse: Internal organization and axon trajectories. J Comp Neurol. 2015 10.1002/cne.23904 . 26400815 PMC4851107

[10] Matsuyama K, Mori F, Kuze B, Mori S. Morphology of single pontine reticulospinal axons in the lumbar enlargement of the cat: A study using the anterograde tracer PHA-L. J Comp Neurol. 1999;410(3):413–30. 10404409

[11] Matsuyama K, Takakusaki K, Nakajima K, Mori S. Multi-segmental innervation of single pontine reticulospinal axons in the cervico-thoracic region of the cat: Anterograde PHA- L tracing study. J Comp Neurol. 1997;377(2):234–50. 8986883

[12] Matsuyama K, Mori F, Nakajima K, Drew T, Aoki M, Mori S. Locomotor role of the corticoreticular–reticulospinal–spinal interneuronal system. 2004;143:239–49. 10.1016/s0079-6123(03)43024-0 14653169

[13] Bannatyne BA, Liu TT, Hammar I, Stecina K, Jankowska E, Maxwell DJ. Excitatory and inhibitory intermediate zone interneurons in pathways from feline group I and II afferents: differences in axonal projections and input. J Physiol. 2009;587(Pt 2):379–99. 10.1113/jphysiol.2008.159129 19047211 PMC2670051

[14] Bareyre FM, Kerschensteiner M, Raineteau O, Mettenleiter TC, Weinmann O, Schwab ME. The injured spinal cord spontaneously forms a new intraspinal circuit in adult rats. Nat Neurosci. 2004;7(3):269–77. 10.1038/nn1195 . 14966523

[15] Stokke MF, Nissen UV, Glover JC, Kiehn O. Projection patterns of commissural interneurons in the lumbar spinal cord of the neonatal rat. Journal of Comparative Neurology. 2002;446(4):349–59. 11954034 10.1002/cne.10211

[16] Quinlan KA, Kiehn O. Segmental, synaptic actions of commissural interneurons in the mouse spinal cord. J Neurosci. 2007;27(24):6521–30. 10.1523/JNEUROSCI.1618-07.2007 . 17567813 PMC6672441

[17] Kiehn O. Locomotor circuits in the mammalian spinal cord. Annu Rev Neurosci. 2006;29:279–306. 10.1146/annurev.neuro.29.051605.112910 . 16776587

[18] Matsuyama K, Nakajima K, Mori F, Aoki M, Mori S. Lumbar commissural interneurons with reticulospinal inputs in the cat: Morphology and discharge patterns during fictive locomotion. Journal of Comparative Neurology. 2004;474(4):546–61. 15174072 10.1002/cne.20131

[19] Jankowska E, Hammar I, Slawinska U, Maleszak K, Edgley SA. Neuronal basis of crossed actions from the reticular formation on feline hindlimb motoneurons. journal of neuroscience. 2003;23(5):1867–78. 12629191 10.1523/JNEUROSCI.23-05-01867.2003 PMC1890022

[20] Bannatyne BA, Edgley SA, Hammar I, Jankowska E, Maxwell DJ. Networks of inhibitory and excitatory commissural interneurons mediating crossed reticulospinal actions. European Journal of Neuroscience. 2003;18(8):2273–84. 14622188 10.1046/j.l460-9568.2003.02973.x PMC1971243

[21] Bolton PS, Goto T, Wilson VJ. Commissural neurons in the cat upper cervical spinal cord. Neuroreport. 1991;2(12):743–6. . 1793815 10.1097/00001756-199112000-00003

[22] Sugiuchi Y, Kakei S, Shinoda Y. Spinal commissural neurons mediating vestibular input to neck motoneurons in the cat upper cervical spinal cord. Neurosci Lett. 1992;145(2):221–4. . 1465221 10.1016/0304-3940(92)90027-5

[23] Alstermark B, Kummel H. Transneuronal transport of wheat germ agglutinin conjugated horseradish peroxidase into last order spinal interneurones projecting to acromio- and spinodeltoideus motoneurones in the cat. 2. Differential labelling of interneurones depending on movement type. Exp Brain Res. 1990;80(1):96–103. . 1694138 10.1007/BF00228851

[24] Soteropoulos DS, Edgley SA, Baker SN. Spinal commissural connections to motoneurons controlling the primate hand and wrist. J Neurosci. 2013;33(23):9614–25. 10.1523/JNEUROSCI.0269-13.2013 23739958 PMC3951829

[25] Brockett EG, Seenan PG, Bannatyne BA, Maxwell DJ. Ascending and descending propriospinal pathways between lumbar and cervical segments in the rat: evidence for a substantial ascending excitatory pathway. Neuroscience. 2013;240:83–97. 10.1016/j.neuroscience.2013.02.039 . 23454541

[26] Ni Y, Nawabi H, Liu X, Yang L, Miyamichi K, Tedeschi A, et al Characterization of long descending premotor propriospinal neurons in the spinal cord. J Neurosci. 2014;34(28):9404–17. 10.1523/JNEUROSCI.1771-14.2014 25009272 PMC4468139

[27] Reed WR, Shum-Siu A, Whelan A, Onifer SM, Magnuson DS. Anterograde labeling of ventrolateral funiculus pathways with spinal enlargement connections in the adult rat spinal cord. Brain Res. 2009;1302:76–84. 10.1016/j.brainres.2009.09.049 19766612 PMC2783768

[28] Juvin L, Simmers J, Morin D. Propriospinal circuitry underlying interlimb coordination in mammalian quadrupedal locomotion. journal of neuroscience. 2005;25(25):6025–35. 15976092 10.1523/JNEUROSCI.0696-05.2005 PMC6724791

[29] Ballion B, Morin D, Viala D. Forelimb locomotor generators and quadrupedal locomotion in the neonatal rat. Eur J Neurosci. 2001;14(10):1727–38. . 11860467 10.1046/j.0953-816x.2001.01794.x

[30] Alstermark B, Lundberg A, Pinter M, Sasaki S. Long C3-C5 propriospinal neurones in the cat. Brain Res. 1987;404(1–2):382–8. . 3032341 10.1016/0006-8993(87)91400-4

[31] Alstermark B, Lundberg A, Pinter M, Sasaki S. Subpopulations and functions of long C3-C5 propriospinal neurones. Brain Res. 1987;404(1–2):395–400. . 3567583 10.1016/0006-8993(87)91402-8

[32] Alstermark B, Isa T, Tantisira B. Integration in descending motor pathways controlling the forelimb in the cat. 18. Morphology, axonal projection and termination of collaterals from C3-C4 propriospinal neurones in the segment of origin. Exp Brain Res. 1991;84(3):561–8. . 1864327

[33] Jankowska E, Edgley SA. How can corticospinal tract neurons contribute to ipsilateral movements? A question with implications for recovery of motor functions. Neuroscientist. 2006;12(1):67–79. 10.1177/1073858405283392 16394194 PMC1890027

[34] Liang FY, Moret V, Wiesendanger M, Rouiller EM. Corticomotoneuronal connections in the rat: evidence from double-labeling of motoneurons and corticospinal axon arborizations. J Comp Neurol. 1991;311(3):356–66. 10.1002/cne.903110306 . 1720143

[35] Brosamle C, Schwab ME. Cells of origin, course, and termination patterns of the ventral, uncrossed component of the mature rat corticospinal tract. J Comp Neurol. 1997;386(2):293–303. . 9295153 10.1002/(sici)1096-9861(19970922)386:2<293::aid-cne9>3.0.co;2-x

[36] McKenna JE, Prusky GT, Whishaw IQ. Cervical motoneuron topography reflects the proximodistal organization of muscles and movements of the rat forelimb: a retrograde carbocyanine dye analysis. J Comp Neurol. 2000;419(3):286–96. . 10723005 10.1002/(sici)1096-9861(20000410)419:3<286::aid-cne2>3.0.co;2-3

[37] Neafsey EJ, Bold EL, Haas G, Hurley-Gius KM, Quirk G, Sievert CF, et al The organization of the rat motor cortex: a microstimulation mapping study. Brain Res. 1986;396(1):77–96. . 3708387 10.1016/s0006-8993(86)80191-3

[38] Paxinos G, Watson C. The rat brain in stereotaxic coordinates. 5th ed Amsterdam; Boston: Elsevier Academic Press; 2005.

[39] Soteropoulos DS, Williams ER, Baker SN. Cells in the monkey ponto-medullary reticular formation modulate their activity with slow finger movements. J Physiol. 2012;590(Pt 16):4011–27. 10.1113/jphysiol.2011.225169 22641776 PMC3476645

[40] Ericson H, Blomqvist A. Tracing of neuronal connections with cholera toxin subunit B: light and electron microscopic immunohistochemistry using monoclonal antibodies. J Neurosci Methods. 1988;24(3):225–35. . 2458510 10.1016/0165-0270(88)90167-7

[41] Chen S, Aston-Jones G. Evidence that cholera toxin B subunit (CTb) can be avidly taken up and transported by fibers of passage. Brain Res. 1995;674(1):107–11. . 7773677 10.1016/0006-8993(95)00020-q

[42] Nyberg-Hansen R, Mascitti TA. Sites and Mode of Termination of Fibers of the Vestibulospinal Tract in the Cat. An Experimental Study with Silver Impregnation Methods. J Comp Neurol. 1964;122:369–83. . 14184860 10.1002/cne.901220307

[43] Wilson VJ, Peterson BW. Peripheral and central substrates of vestibulospinal reflexes. Physiol Rev. 1978;58(1):80–105. . 341186 10.1152/physrev.1978.58.1.80

[44] Petras JM. Cortical, tectal and tegmental fiber connections in the spinal cord of the cat. Brain Res. 1967;6(2):275–324. . 6060511 10.1016/0006-8993(67)90196-5

[45] Shinoda Y, Ohgaki T, Sugiuchi Y, Futami T. Morphology of single medial vestibulospinal tract axons in the upper cervical spinal cord of the cat. J Comp Neurol. 1992;316:151–72. 1573053 10.1002/cne.903160203

[46] Angelucci A, Clascá F, Sur M. Anterograde axonal tracing with the subunit B of cholera toxin: A highly sensitive immunohistochemical protocol for revealing fine axonal morphology in adult and neonatal brains. J Neurosci Meth. 1996;65(1):101–12. 10.1016/0165-0270(95)00155-7 8815303

[47] Todd AJ. A method for combining confocal and electron microscopic examination of sections processed for double- or triple-labelling immunocytochemistry. J Neurosci Methods. 1997;73(2):149–57. 9196286 10.1016/s0165-0270(97)02222-x

[48] Olave MJ, Maxwell DJ. Neurokinin-1 projection cells in the rat dorsal horn receive synaptic contacts from axons that possess α2C-adrenergic receptors. journal of neuroscience. 2003;23(17):6837–46. 12890778 10.1523/JNEUROSCI.23-17-06837.2003 PMC6740721

[49] Stepien AE, Tripodi M, Arber S. Monosynaptic rabies virus reveals premotor network organization and synaptic specificity of cholinergic partition cells. Neuron. 2010;68(3):456–72. 10.1016/j.neuron.2010.10.019 . 21040847

[50] Alstermark B, Isa T, Tantisira B. Pyramidal excitation in long propriospinal neurones in the cervical segments of the cat. Exp Brain Res. 1991;84(3):569–82. . 1864328 10.1007/BF00230969

[51] Alstermark B, Sasaki S. Integration in descending motor pathways controlling the forelimb in the cat. 13. Corticospinal effects in shoulder, elbow, wrist, and digit motoneurones. Exp Brain Res. 1985;59(2):353–64. . 4029309 10.1007/BF00230915

[52] Alstermark B, Ogawa J, Isa T. Lack of monosynaptic corticomotoneuronal EPSPs in rats: disynaptic EPSPs mediated via reticulospinal neurons and polysynaptic EPSPs via segmental interneurons. J Neurophysiol. 2004;91(4):1832–9. 10.1152/jn.00820.2003 . 14602838

[53] Huisman AM, Kuypers HG, Verburgh CA. Quantitative differences in collateralization of the descending spinal pathways from red nucleus and other brain stem cell groups in rat as demonstrated with the multiple fluorescent retrograde tracer technique. Brain Res. 1981;209(2):271–86. . 7225794 10.1016/0006-8993(81)90153-0

[54] Cavada C, Huisman AM, Kuypers HG. Retrograde double labeling of neurons: the combined use of horseradish peroxidase and diamidino yellow dihydrochloride (DY X 2HCl) compared with true blue and DY X 2HCl in rat descending brainstem pathways. Brain Res. 1984;308(1):123–36. . 6548167 10.1016/0006-8993(84)90923-5

[55] Sivertsen MS, Glover JC, Perreault MC. Organization of pontine reticulospinal inputs to motoneurons controlling axial and limb muscles in the neonatal mouse. J Neurophysiol. 2014;112(7):1628–43. 10.1152/jn.00820.2013 . 24944221 PMC4631545

[56] Zaaimi B, Edgley SA, Soteropoulos DS, Baker SN. Changes in descending motor pathway connectivity after corticospinal tract lesion in macaque monkey. Brain. 2012;135(Pt 7):2277–89. 10.1093/brain/aws115 22581799 PMC3381720

[57] Bachmann LC, Lindau NT, Felder P, Schwab ME. Sprouting of brainstem-spinal tracts in response to unilateral motor cortex stroke in mice. J Neurosci. 2014;34(9):3378–89. 10.1523/JNEUROSCI.4384-13.2014 . 24573294 PMC6795311

