# Correction: Corticospinal and Reticulospinal Contacts on Cervical Commissural and Long Descending Propriospinal Neurons in the Adult Rat Spinal Cord; Evidence for Powerful Reticulospinal Connections

**DOI:** 10.1371/journal.pone.0155664

**Published:** 2016-05-11

**Authors:** Emma J. Mitchell, Sarah McCallum, Deborah Dewar, David J. Maxwell

The following information is missing from the Funding section: This work was funded by the Medical Research Council (grant number MR/J50032X/1).
